# The position with fingers on clavicles has no influence on vertical trunk inclination and kyphosis but significantly changes surface topography lordosis angle

**DOI:** 10.1186/1748-7161-8-S2-O21

**Published:** 2013-09-18

**Authors:** Glinkowski Wojciech, Izabela Czyżak, Bożena Glinkowska, Agnieszka Żukowska, Michoński Jakub

**Affiliations:** 1 Chair and Department of Orthopaedics and Traumatology of Locomotor System, Center of Excellence “TeleOrto”, Baby Jesus Clinical Hospital, Medical University of Warsaw, Poland

## Background

Radiographic lateral spine view remains the gold-standard evaluation for sagittal curvatures of the spine. Specific positioning of the forward upper extremities during radiographic exposure is recommended to ensure no vertebra is obscured. However, the postural impact of arm positions has never been verified for the normal adolescent population. As a result, no data are available about the influence of arms positioning on the sagittal positioning of the trunk.

## Purpose

The aim of this study is to measure the effect of arm positioning on surface topography measures.

## Methods

In a cross-sectional study, 694 subjects (K=412; F=275) aged 10-18 years of age were examined and were scanned with a 3-dimensional (3D) telediagnostic system for postural screening. For this study, we used 3D surface topography (“3D Orthoscreen”) enhanced by structured light and accurate calibration in XYZ Cartesian space, which enables the system to measure vertical trunk inclination angle against the plumb line. The statistical analysis was performed using SAS v.9.3.

## Results

No systematic difference was found between measurements in two different positions. British Standardization Institute Index (BSII) (2*SD) was 11.4°. Results for 94.2% of the subjects fulfilled the criteria for BSII. Reliability index (ICC) was 0.60; p<0.0001; higher for girls (0.65) than for boys (0.5). We discovered systematic differences between lordosis angle values in standing positions with hands hanging freely and “fingers on the clavicles”. The average value of standing position with flexed elbows was higher. Observed differences were significant regardless of gender. The correlation between results in two positions was 0.86 in girls and 0.8 in boys. No systematic differences were found for trunk vertical inclination in sagittal plane in relation to two different positions. BSII was (2*SD) 4.84. Results for 94.2% of the subjects fulfilled the criteria for BSII. The reliability intraclass correlation coefficients index represented a significant correlation between results 0.74; p<0.0001, slightly higher for girls (0.78) than for boys (0.72).

## Conclusions and discussion

We conclude that the habitual position, regardless of elbow extension or flexion, has no influence on sagittal thoracic curvature, and on vertical trunk inclination against plumb line. However, the position with fingers on the clavicles significantly influences lumbar lordosis. The 3D surface topography may help when considering the real habitual standing angles.
